# Dr. George Miles Palmer

**Published:** 1905-07

**Authors:** 


Dr. George Miles Palmer, a graduate of the University of Buffalo, 1865, died at his home at Warsaw, N. Y., May 28, 1905, aged 77 years. He was a native of Angelica, but lived and practised his profession for many years at Pike, of which town he was supervisor for five terms. He was also a medical officer in the army during the civil war. He represented Wyoming county in the legislature for one term. In 1891, Dr. Palmer removed to Warsaw, of which village he has served as health officer and he was also a U. S. pension examining surgeon.

